# Utility of Nuclear Morphometry in Predicting Grades of Diffusely Infiltrating Gliomas

**DOI:** 10.1155/2013/760653

**Published:** 2013-08-26

**Authors:** Dibyajyoti Boruah, Prabal Deb

**Affiliations:** Department of Pathology, Armed Forces Medical College, Pune, Maharashtra 411040, India

## Abstract

*Introduction*. The ability to reliably differentiate neoplastic from nonneoplastic specimen and ascertain the tumour grade of diffusely infiltrating gliomas (DIGs) is often challenging. *Aims and Objective*. To evaluate utility of image morphometry in identifying DIG areas and to predict tumour grade. *Materials and Methods*. Image morphometry was used to analyze the following nuclear features of 30 DIGs and 10 controls (CG): major axis of nucleus (MAJX), minor axis of nucleus (MINX), nuclear area (NA), nuclear perimeter (NP), nuclear roundness (NR), nuclear density (ND), and percentage of total nuclear area (%TNA). *Results*. Statistically significant differences in all parameters, except NR, were observed between all groups, with strong positive correlation with tumour grade (*r* > 0.7). The mean values were maximum for HGG and minimum for CG. For NR, the difference between CG/HGG was statistically significant, unlike CG/LGG and LGG/HGG. It was observed that NA distributions for CG were nearly Gaussian type with smaller range, while gliomas displayed erratic pattern with larger range. NA and NP exhibited strong positive correlation with ND. *Conclusion*. Image morphometry has immense potential in being a powerful tool to distinguish normal from neoplastic tissue and also to differentiate LGG from HGG cases, especially in tiny stereotactic biopsies.

## 1. Introduction 

The global annual incidence of primary malignant central nervous system (CNS) tumors is around 37 per million for male and 26 per million for female [1, 2], with gliomas constituting the majority.

In the past several years, it has been well established that quite a few clinical and histopathological parameters are helpful in predicting the clinical outcome of cancer patients. Currently, well-established techniques like morphometry, stereology, static image, and flow cytometry are routinely used in diagnostic quantitative pathology. The potential significance of these techniques includes the objective distinction between benign, borderline, and malignant lesions; the objective grading of invasive tumors; and the prediction of prognosis and therapeutic response. 

Computer-assisted image analysis is a new powerful tool for high-precision measurement of different facets of tumor cells to achieve similar goals [3–7]. To date only few studies have utilized nuclear morphometric measurements, like mean major axis (MAJX), minor axis (MINX), nuclear area (NA), nuclear perimeter (NP), and roundness of nucleus (NR), to determine the nuclear size and shape profiles in neoplastic tissues in CNS tumors [6–12]. The initial four parameters, namely, MAJX, MINX, NA, and NP, are related to nuclear size (hypertrophy), while NR is related to nuclear shape.

In the present study, mean nuclear density (ND) and percentage of total nuclear area (%TNA) were also evaluated to analyze their utility in the gliomas. These two parameters are related to the growth rate of the tumor. The main objectives of this study were to correlate morphometrically measured MAJX, MINX, NA, NP, NR, ND, and %TNA with WHO grade of diffusely infiltrating gliomas (DIG); and to assess their ability to differentiate normal from neoplastic areas.

## 2. Materials and Methods

The present study included biopsies from thirty cases of DIGs that were managed at this tertiary care institute during 2009–2011. The spectrum of cases included (a) 14 cases of low-grade gliomas [LGG: ten diffuse astrocytoma (DA: WHO grade-II), four oligoastrocytoma (OA: WHO grade-II)] and (b) 16 cases of high-grade gliomas [HGG: thirteen glioblastoma multiforme (GBM: WHO grade-IV), three anaplastic astrocytoma (AA: WHO grade-III)]. Brain tissue from ten autopsy cases (three of ischemic stroke; two of haemorrhagic stroke; and five without any gross or microscopic brain pathology) [13] without CNS tumours, on histology, constituted the controls.

The mean age at surgery was 39.1 years (range: 9–62 years) for LGG and 54.6 years (range: 27–79 years) for HGG. There was only one case in the pediatric age (<18 years) group (low grade astrocytoma).

### 2.1. Nuclear Morphometry

**Figure 1:**
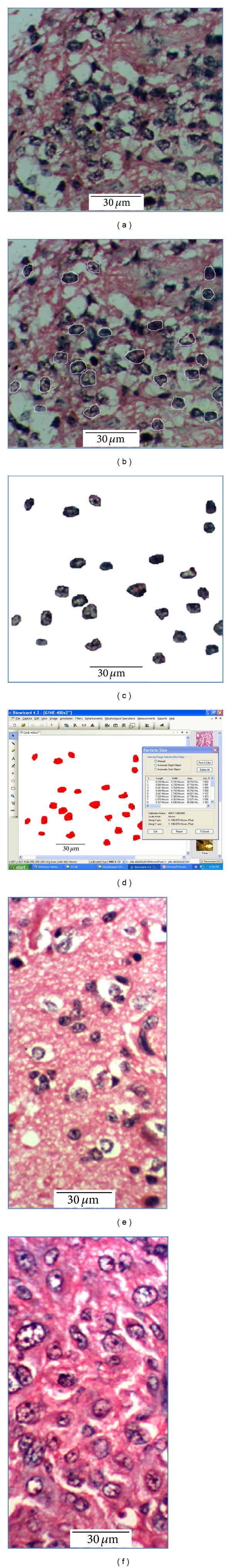
(a) Image of an HE-stained section; (b) nuclei were outlined for morphometry; (c) outlined nuclei were separated; (d) screenshot demonstrating the measurement of nuclear parameters using image analysis software. Representative images of HE-stained sections: (e) low-grade glioma and (f) high-grade glioma.

Morphometric analysis was performed on hematoxylin and eosin (H&E)-stained histological sections of 5-micron thickness of formalin fixed paraffin-embedded CNS tissue, having optimal histological detail. A computerized digital photomicrograph system (Dewinter Optical Inc. with Digi Eye 330 digital photomicrography camera and Biowizard 4.2 Image analysis software) was used for image analysis. The measuring scale of the image analysis software was properly calibrated. For each sample, five high power fields (400x), having maximum cellularity, were recorded for the study. For each case, 100 nuclei, clearly separated from others and relatively larger in size, were chosen to evaluate nuclear shape and size. The nuclei were outlined using a mouse attached to the computer and then separated from others prior to the determination of their nuclear parameters using the software (Figure 1). After measurement, the data has been transferred to an MS-Excel sheet for further analysis. Nuclei were analyzed for major axis (MAJX), minor axis (MINX), nuclear area (NA), nuclear perimeter (NP), and nuclear roundness (NR). The nuclear roundness is expressed as [(4*π*·nuclear area)/(nuclear perimeter)^2^] × 100 in percentage. Thus, for a perfectly round nucleus its value is maximum, that is, equal to 100. A nucleus having irregular shape had smaller value of NR.

Counting of nucleus was done in the five recorded high power fields from which the mean nuclear density (ND) was calculated. The percentage of total nuclear area (%TNA) was determined for the five high power fields for each sample using the software, and their mean was calculated.

### 2.2. Statistical Analysis

The various parameters used to analyze were MAJX, MINX, NA, NP, and NR for each nucleus of every sample of the three groups. Mean ND and %TNA were determined for each sample. The mean values of these parameters with standard deviation (SD) and range were calculated for the three groups: CG, LGG, and HGG. Student's *t*-test was performed to evaluate the difference in MAJX, MINX, NA, NP, NR, ND, and %TNA for each pair of the groups, and the *P* values were determined. Data was reported as mean, standard deviation of mean, and range of mean for these parameters. The statistical correlations of the analyzed morphometric parameters with tumor grades of all samples were investigated; correlations of ND with NA and NP were also studied. Pearson correlation coefficient (“*r*”) and *P*  value were calculated, and regression line was drawn in correlation studies.

The distribution of the measured area of nucleus for five samples of each group is also shown along with the calculated Gaussian distribution of nuclear area using mean and SD of the respective group. 

## 3. Results 

**Figure 2:**
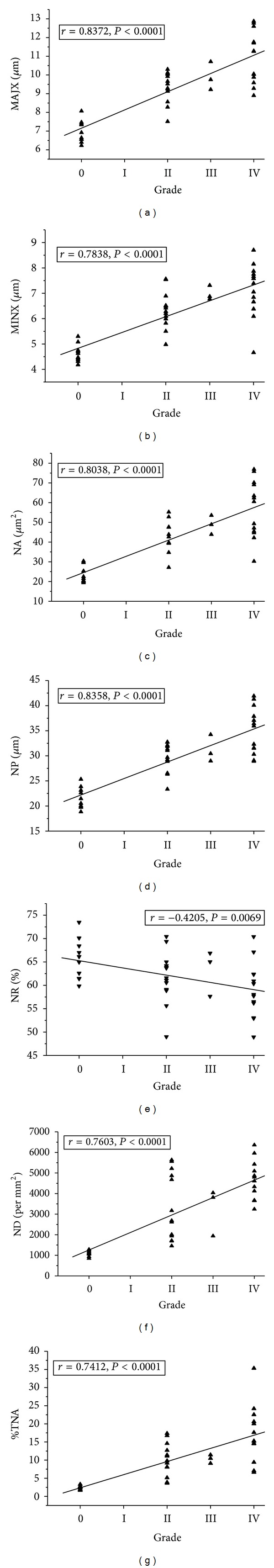
Scatter plot of grades versus (a) MAJX, (b) MINX, (c) NA, (d) NP, (e) NR, (f) ND, and (g) %TNA for all study and control samples: linear regression between these parameters and grades is shown by the solid lines in their respective plot.

Figures 2(a)–2(g) represent the correlation of the mean values of MAJX, MINX, NA, NP, NR, ND, and %TNA of each sample with WHO grade (2007 classification), respectively. In case of controls, the grade was considered as 0. The parameters: MAJX, MINX, NA, NP, ND, and %TNA, showed strong positive correlation with tumour grade (*r* > 0.70), whereas NR demonstrated mild negative correlation with grade (*r* = −0.42). 

**Figure 3:**
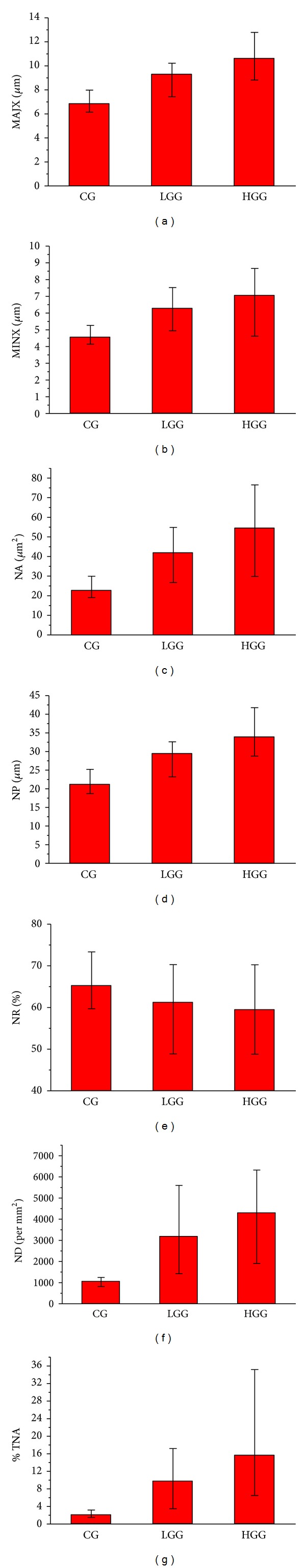
Mean value and range (error bar) of (a) MAJX, (b) MINX, (c) NA, (d) NP, (e) NR, (f) ND, and (g) %TNA for the two study (LGG & HGG) and control (CG) groups.

The mean values of nuclear parameters and age at the time of surgery with standard deviation (SD) and range for controls, LGG, and HGG are presented in Table 1. The *P*  values to predict the difference of each pair of these three groups for all the parameters are also presented in this table. The mean values of MAJX, MINX, NA, NP, NR, ND, and %TNA of the three groups were represented in Figures 3(a)–3(g) respectively; the range of the parameters for each group was presented as error bar in the figures.

It was noted that the nuclear parameters: mean MAJX, MINX, NA, NP, ND, and %TNA, of the two tumor groups, LGG and HGG were significantly greater than the controls (*P* < 0.0004) and that NR in HGG was significantly smaller than the controls (*P* = 0.0125). Again, between two tumor groups, age, MAJX, MINX, NA, NP, ND, and %TNA in HGG were significantly greater than LGG (*P* < 0.05), unlike NR (*P* = 0.4011).

### 3.1. Controls (CG)

The mean age of the CG was 46.3 years (range: 40–53 years). Nuclei, in the control tissues, were evenly distributed, with mean MAJX: 6.96 *µ*m (range: 6.23 *µ*m–8.07 *µ*m), mean MINX: 4.62 *µ*m (range: 4.18 *µ*m–5.30 *µ*m), mean NA: 23.44 *µ*m^2^ (range: 19.42 *µ*m^2^–30.33 *µ*m^2^), mean NP: 21.54 *µ*m (range: 18.84 *µ*m–25.33 *µ*m), and mean NR: 65.55 (range: 59.85–73.48). ND was 1107 mm^−2^ (range: 849 mm^−2^–1282 mm^−2^), while mean %TNA was 2.32 (range: 1.65–3.33).

### 3.2. Low-Grade Gliomas (LGG)

The mean age of LGGs was 39.1 years (range: 9–62 years). In LGG, the following was noted mean MAJX was 9.41 *µ*m (range: 7.51 *µ*m–10.30 *µ*m); mean MINX was 6.34 *µ*m (range: 4.98 *µ*m–7.56 *µ*m); and mean NA was 42.62 *µ*m^2^ (range: 27.15 *µ*m^2^–55.23 *µ*m^2^); mean NP was 29.79 *µ*m (range: 23.34 *µ*m–32.74 *µ*m); and mean NR was 61.53 (range: 48.99–70.43). ND was 3232 mm^−2^ (range: 1456 mm^−2^–5633 mm^−2^), and mean %TNA was 9.98 (range: 3.63–17.35). MAJX, MINX, NA, NP, ND, and %TNA in LGG were significantly larger than controls (*P* < 0.0004) but significantly smaller than the HGG (*P* < 0.05). Age of patients was also found significantly lower in LGG as compared to HGG (*P* = 0.0116).

### 3.3. High-Grade Gliomas (HGG)

The mean age of the HGG was 54.6 years (range: 27–79 years). In this group the following was noted: mean MAJX was 10.73 *µ*m (range: 8.90 *µ*m–12.87 *µ*m); mean MINX was 7.11 *µ*m (range: 4.66 *µ*m–8.70 *µ*m); mean NA was 55.22 *µ*m^2^ (range: 30.26 *µ*m^2^–76.90 *µ*m^2^); mean NP was 34.24 *µ*m (range: 28.92 *µ*m–41.91 *µ*m); and mean NR was 59.78 (range: 48.94–70.40). ND was 4350 mm^−2^ (range: 1941 mm^−2^–6360 mm^−2^) and mean %TNA was 15.88 (range: 6.64–35.33). MAJX, MINX, NA, NP, ND, and %TNA in LGG were significantly larger than controls (*P* < 0.0001), and LGG (*P* < 0.02). In HGG, the mean age was also found significantly higher than LGG (*P* = 0.0116), and mean NR was significantly lower than controls (*P* = 0.0125).

### 3.4. Area Distribution of Nuclei

**Figure 4:**
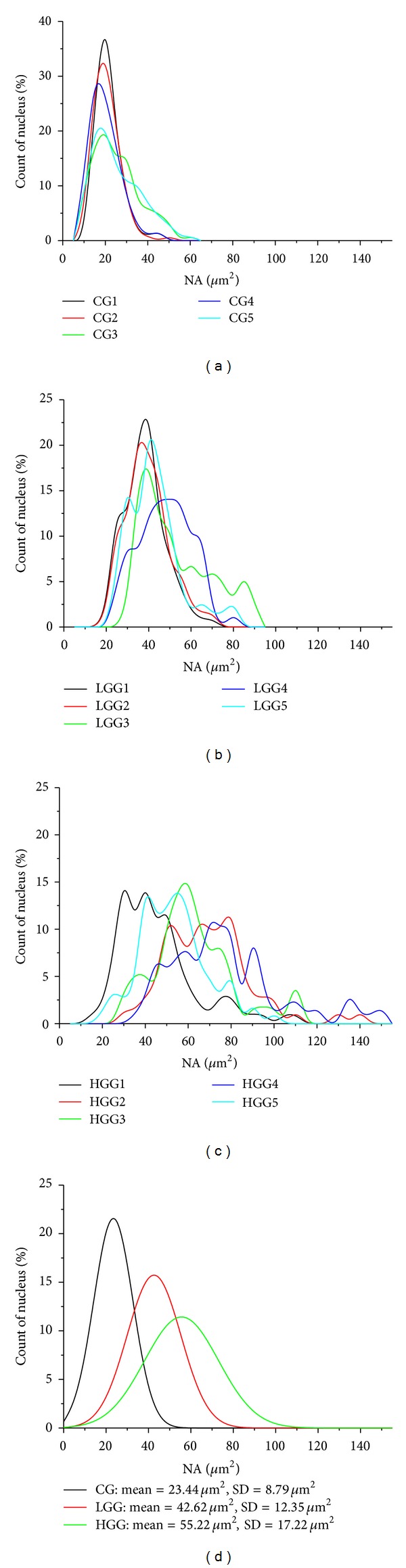
The measured distribution of nuclear area for (a) five samples (CG1, CG2, CG3, CG4, and CG5) of the controls (CG); (b) five samples (LGG1, LGG2, LGG3, LGG4, and LGG5) of the low-grade gliomas (LGG); (c) for five samples (HGG1, HGG2, HGG3, HGG4, and HGG5) of the high grade gliomas (HGG); (d) Calculated Gaussian distribution of nuclear area for CG, LGG, and HGG groups using their respective mean nuclear area (NA) and standard deviation (SD) for number of nuclei *n* = 100.

Figures 4(a)–4(d) represent distribution of nuclear area. Figures 4(a), 4(b), and 4(c) represent the measured distribution of nuclear area of CG, LGG, and HGG for five samples of each group. It was observed that the distributions for the normal samples were nearly Gaussian with smaller range, but in tumors, the distributions deviated from Gaussian pattern and displayed larger range and smaller height than the controls.  Figure 4(d) shows the predicted Gaussian distribution of nuclear area for CG, LGG, and HGG, using mean NA and mean SD of NA of respective group.

### 3.5. Correlation of ND with NA and NP

**Figure 5:**
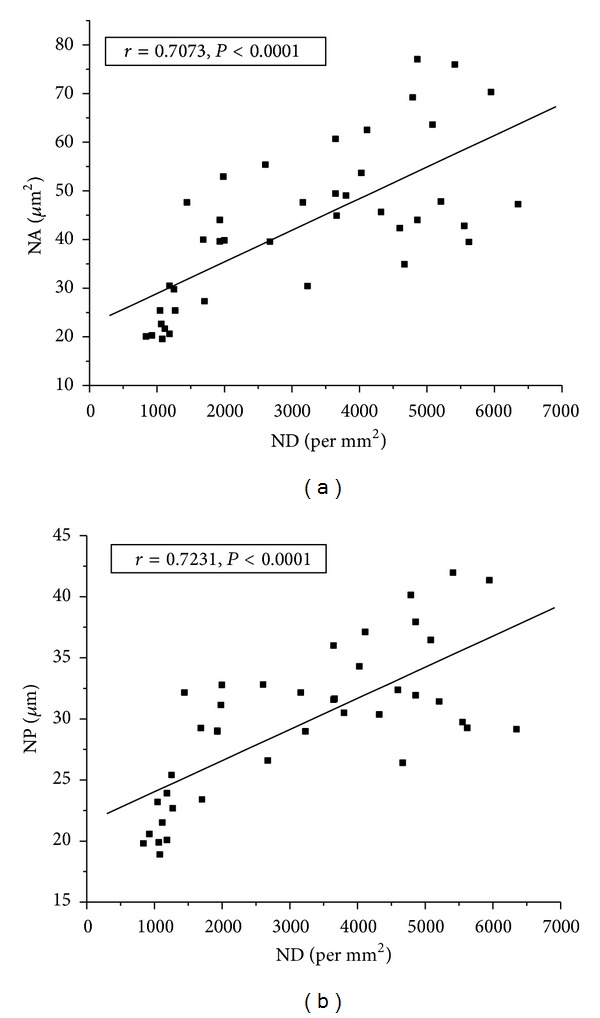
Scatter plot of ND versus (a) NA and (b) NP for all study and control samples: linear regression between these parameters and grades is shown by the solid lines in their respective plot.

Figures 5(a) and 5(b) represent scatter plots of ND versus NA and NP for all study and control samples with linear regressions. Both NA and NP showed strong positive correlation with ND, but NP exhibited better correlation (*r* = 0.72) than NA (0.70).

## 4. Discussion 

Nuclear morphometric features have a potential role in differentiating between the different grades of gliomas and predicting patient survival [6–8]. Niedermayer et al. studied nuclear area percentage, nuclear size, and pleomorphism in gliomas. Using the regression analysis of survival data, they observed that the best predictors for patient outcome were grading, as observed on histology, and nuclear area percentage, as evaluated by image analysis [12]. Pennella et al. reported that the shape factor in conjunction with nuclear pleomorphism and mitotic activity might possess a better ability to distinguish atypical and malignant meningiomas [10]. In a recent study on meningiomas, Noy et al. described that tumors with less nuclear orientation, more nuclear density, higher fractal dimension, and less regular chromatin textures had a tendency to recur faster [9].

In the present study, we observed that in cases of CNS lesions, a panel of nuclear parameters evaluated by image morphometry was not only able to reliably distinguish normal from neoplastic areas, but also to indicate consistently the grade of tumour. Nuclei of astrocytic cells in the control groups had the smallest value of MAJX, MINX, NA, NP, and ND and %TNA, while neoplastic astrocytes of HGG measured the largest. In cases of parameters related to size of the nucleus, namely, MAJX, MINX, NA, and NP, a strong positive correlation with tumour grades (*r* > 0.78) could be observed, which is translated as follows: *the bigger the size of the nucleus, the higher is its probability of being a glioma of higher grade*. Of these, NP and MAJX displayed the best correlation (*r* = 0.83).

ND and %TNA, parameters related to the rate of growth of a tumor, showed strong positive correlation with tumour grade (*r* > 0.74), thus confirming that *higher grade tumors are indeed more susceptible to a rapid rate of growth*. The faster growth rate in higher-grade tumor leads to an increase in the number density of nucleus, which is histologically seen as closely packed tumor cells with higher percentage of tumour area occupied by dysplastic nuclei. 

In the current study, NA and NP exhibited strong positive correlation with ND, with NP being better. It is accepted that in high-grade tumors the nutritional and oxygen demands per unit volume of tissue are higher, which it tries to compensate by stimulating various proangiogenic factors like hypoxia inducible factor (HIF-1*α*) that enhances the microvascular networks in the tumour [5]. However, the increased nutritional and oxygen requirements per unit volume of tissue in HGG may not always be fulfilled by the increased microvascular network. *Therefore, it can be hypothesized that the nuclei of the tumour cells enlarge in an effort to increase their surface area so as to enhance the diffusion rate to maintain their required metabolic function. *In support of this, it was observed that NP directly related to the surface area of a nucleus in three-dimensional cases, showed the best correlation with the tumour grade and nuclear density.

The nuclear roundness, which was related to shape of the nuclei, showed a mild negative correlation with tumour grade (*r* = −0.42). Hence, the nuclear size and parameters related to tumor growth have more importance in comparison to the parameters related to nuclear shape, for assessment of tumour grade by a quantitative morphometric method. Owing to its high nuclear density, all the nuclei of a closely packed high-grade tumour may not receive sufficient nutrients. *In an effort to compensate there is enhancement in the rate of diffusion, which the tumour cell performs by escalating its surface area per unit nuclear mass, a feature that is seen histologically by the severe pleomorphism in shape of the cells (i.e., deformity in its shape from ideal spherical one). * Owing to this, the value of NR showed negative correlation with grades. 

The nuclear area distributions were nearly Gaussian pattern for the control group, while they deviated from the Gaussian pattern in case of tumours, a feature that can be potentially applied to reliably differentiate neoplastic from normal areas in cytology and in evaluation of stereotactic biopsies. *This may possibly be a resultant of the random multiplication of tumor cells.* It was noted that there was a decrease in the height of the distribution and an increase in the range of the distribution with an increase in tumour grade (Figures 4(a)–4(c)), as expected from their hypothetical Gaussian distribution (Figure 4(d)). The nuclear area distribution pattern may be useful for the classification of tumor grade in diffusely infiltrating gliomas. 

In conclusion, our study showed a definitive correlation between various nuclear morphometric parameters with tumor grade in diffusely infiltrating gliomas. The nuclear morphometric study can be gainfully utilized not only to distinguish normal from neoplastic tissue, but also to differentiate between the various grades of gliomas. It also serves as an excellent model to comprehend various facets of tumour and tumour cell physiology. The present study also revealed that the tumor grade had a better correlation with nuclear enlargement than with nuclear roundness, where MAJX and NP showed the best correlation with grades (*r* = 0.83). The nuclear enlargement showed strong correlation with nuclear density in gliomas. The nuclear area distribution pattern may also be gainfully utilized for identification of different grades of gliomas. These features have potentially immense utility in diagnostically challenging scenarios, like intraoperative cytological specimen, and in the analysis of tiny specimen as in stereotactic biopsies. 

## Figures and Tables

**Table 1:** Mean values of age and nuclear parameters of the two study and the control groups with their standard deviation and range. *P* values between each pair of group for all the parameters.

Parameters (unit) ± SD (min–max)	Group (no. of cases)			*P* value		
	CG (10)	LGG (14)	HGG (16)	*P* (CG versus LGG)	*P* (CG versus HGG)	*P* (LGG versus HGG)
Age (year) ± SD (min–max)	46.3 ± 3.5 (40–53)	39.1 ± 14.1 (9–62)	54.6 ± 16.8 (27–79)	0.1335*	0.1397*	0.0116

MAJX (*μ*m) ± SD (min–max)	6.96 ± 0.59 (6.23–8.07)	9.41 ± 0.81 (7.51–10.30)	10.73 ± 1.33 (8.90–12.87)	<0.0001	<0.0001	0.0032

MINX (*μ*m) ± SD (min–max)	4.62 ± 0.35 (4.18–5.30)	6.34 ± 0.70 (4.98–7.56)	7.11 ± 0.94 (4.66–8.70)	<0.0001	<0.0001	0.0184

NA (*μ*m^2^) ± SD (min–max)	23.44 ± 4.01 (19.42–30.33)	42.62 ± 7.26 (27.15–55.23)	55.22 ± 13.48 (30.26–76.90)	<0.0001	<0.0001	0.0042

NP (*μ*m) ± SD (min–max)	21.54 ± 2.11 (18.84–25.33)	29.79 ± 2.80 (23.34–32.74)	34.24 ± 4.49 (28.92–41.91)	<0.0001	<0.0001	0.0034

NR (%) ± SD (min–max)	65.55 ± 4.34 (59.85–73.48)	61.53 ± 5.42 (48.99–70.43)	59.78 ± 5.80 (48.94–70.40)	0.0659*	0.0125	0.4011*

ND (per mm^2^) ± SD (min–max)	1107 ± 138 (849–1282)	3232 ± 1596 (1456–5633)	4350 ± 1089 (1941–6360)	<0.0004	<0.0001	0.0314

%TNA	2.32 ± 0.54 (1.65–3.33)	9.98 ± 4.34 (3.63–17.35)	15.88 ± 7.48 (6.64–35.33)	<0.0001	<0.0001	0.0151

CG: control group; LGG: low-grade gliomas; HGG: high-grade gliomas; min: minimum; max: maximum; *P*(X versus Y): *P* value between X group versus Y group by Student's unpaired *t*-test; ∗: difference is not significant.
